# Supplementary material for: Global hypo-methylation in a proportion of glioblastoma enriched for an astrocytic signature is associated with increased invasion and altered immune landscape
Source: eLife. 2022 Nov 22;11:e77335. doi: 10.7554/eLife.77335 (PMC9681209; doi:10.7554/eLife.77335)
Supplement: Figure 2—source data 1. [file elife-77335-fig2-data1.zip › Figure_2_source_data_1/Figure_2A/homerResults/motif29.similar.html]

motif29

## Information for motif29

A
C
T
G
A
C
G
T
A
T
C
G
C
G
T
A
T
A
C
G
A
C
G
T
A
C
T
G
T
G
A
C
A
T
G
C
T
G
C
A
  
Reverse Opposite:  

A
C
G
T
A
T
C
G
A
C
T
G
A
G
T
C
C
G
T
A
A
T
G
C
G
C
A
T
A
T
G
C
G
T
C
A
A
G
T
C
  

|  |  |
| --- | --- |
| p-value: | 1e-17 |
| log p-value: | -4.030e+01 |
| Information Content per bp: | 1.798 |
| Number of Target Sequences with motif | 33.0 |
| Percentage of Target Sequences with motif | 1.42% |
| Number of Background Sequences with motif | 7.9 |
| Percentage of Background Sequences with motif | 0.22% |
| Average Position of motif in Targets | 96.2 +/- 56.9bp |
| Average Position of motif in Background | 95.4 +/- 42.8bp |
| Strand Bias (log2 ratio + to - strand density) | -0.1 |
| Multiplicity (# of sites on avg that occur together) | 1.06 |
| Motif File: | file (matrix) reverse opposite |

### Similar de novo motifs found

|  |  |  |  |  |  |  |  |
| --- | --- | --- | --- | --- | --- | --- | --- |
| Rank | Match Score | Redundant Motif | P-value | log P-value | % of Targets | % of Background | Motif file |
| 1 | 0.625 | A C T G C G T A C T A G C G T A A C T G C G T A C A T G C G T A T A G C A T G C | 1e-16 | -38.579801 | 0.69% | 0.04% | motif file (matrix) |
| 2 | 0.737 | A C T G A G T C A C T G A C G T A C T G A G T C A G T C C G T A | 1e-6 | -13.876014 | 0.43% | 0.07% | motif file (matrix) |
